# HMGB3 is a Potential Therapeutic Target by Affecting the Migration and Proliferation of Colorectal Cancer

**DOI:** 10.3389/fcell.2022.891482

**Published:** 2022-05-31

**Authors:** Wenjing Gong, Yang Guo, Hang Yuan, Xinye Hu, Rui Chai, Boan Zheng, Ziang Wan, Shiliang Tu

**Affiliations:** General Surgery, Cancer Center, Department of Colorectal Surgery, Zhejiang Provincial People’s Hospital, Affiliated People’s Hospital of Hangzhou Medical College, Hangzhou, China

**Keywords:** HMGB3, proliferation, migration, CRC, bioinformactics

## Abstract

Colorectal cancer is one of the common malignant tumors in the digestive system, with high incidence and mortality rate. Therefore, there is an urgent need to identify and develop new molecular targets for colorectal cancer treatment. Previous studies have pointed out the important role of HMGB3 in tumors, and how it works in colorectal cancer needs to be studied in depth. In this study, we found that HMGB3 was highly expressed in COAD in the cBioPortal and GEPIA2 databases. Kaplan-Meier analysis showed that compared with patients with lower HMGB3 levels, patients with higher HMGB3 levels had poorer OS (*p* = 0.001). We also found a correlation between HMGB3 expression and immune infiltration of CRC. To investigate the mechanism of HMGB3 knockdown-mediated colorectal cancer inhibition, we detected a downregulation of N-cadherin, Vimentin and β-catenin proteins after knockdown of HMGB3. Taken together, HMGB3 can be an effective target for CRC treatment in the future, and we have reason to believe that HMGB3 will be of greater value in more tumors in the near future.

## Introduction

Colorectal cancer is one of the common malignant tumors in the digestive system (Song et al., 2015), with high incidence and mortality rate (Arnold et al., 2017), especially the five-year survival rate of advanced colorectal cancer is low (Tarazi et al., 2018). Although there are various treatment modalities available for colorectal cancer, including preoperative neoadjuvant radiotherapy (Adam et al., 2015), surgical resection of the tumor (Akgül et al., 2014) and postoperative adjuvant radiotherapy (Shuwen et al., 2020), as well as current targeted immunotherapy (Ganesh et al., 2019). Due to the characteristics of colorectal cancer such as easy metastasis through blood and direct invasion of adjacent organs, the treatment of advanced colorectal cancer is ineffective. Therefore, there is an urgent need to identify and develop new molecular targets for colorectal cancer treatment. Molecular markers may not only provide the basis for diagnosis, but also may provide therapeutic targets for clinical treatment (Ganesh et al., 2019).

High mobility group box3 (hmgb3) belongs to the high mobility group protein family. It plays an important role in DNA replication and transcription (Petit et al., 2010). It is a pathogenic gene of leukemia, and it is a marker involved in tumorigenesis and development by regulating cell cycle (Tang et al., 2012). It is a kind of genes that are highly expressed in tumors and promote the occurrence and development of tumors. For example, HMGB3 can be regulated by ncRNAs, such as those involved in tumor progression and development (Sun et al., 2021). HMGB3 has also been shown to be involved in the regulation of breast cancer cell proliferation by inhibiting the β-catenin signaling pathway (Zhuang et al., 2020). Previous studies have pointed out the important role of HMGB3 in tumors, and how it works in colorectal cancer needs to be studied in depth.

In this study, we investigated the expression and prognosis of HMGB3 in colorectal cancer patients in the cBioPortal and GEPIA2 databases. In addition, we analyzed the methylation of HMGB3 and other profiles to explore the potential mechanisms of HMGB3 in colorectal cancer. We explored the expression abundance of HMGB3 in colorectal cancer cell lines. We also constructed an siRNA plasmid as well as an overexpression plasmid of HMGB3 to verify the mechanism of HMGB3 in colorectal cancer by *in vitro* cytological assays. Our results suggest that HMGB3 is correlated with the proliferation and migration of colorectal cancer cells and can be a possible new prognostic indicator and therapeutic target for CRC patients.

## Materials and Methods

### Dataset Analyses

GEPIA2 databases: GEPIA2 is an updated version of GEPIA for analysis of = RNA sequencing expression data from 9736 tumor and 8587 normal samples from TCGA and GTEx projects. The key added features add analysis of genetic subtypes, classification of tumor subtypes, and the ability for users to upload their own data for comparison with data on TCGA and GTEx, blending.

cBioPortal: The cBioPortal website integrates data from 126 cancer genomic studies, including large cancer esearch projects such as TCGA and ICGC, covering data from 28,000 specimens, in addition to some samples containing phenotypic information such as clinical prognosis. the OncoPrint page displays the relationship between continuous and discrete values of genes in the form of scatter plots, while the mutation page displays the relationship between continuous and discrete values in the form of structured graphs display mutation information for multiple samples, including site, type, amino acid changes, etc. The Co-expression page displays genes that are co-expressed with the retrieved genes, as well as Pearson and Spearman correlation coefficients. The Enrichment page focuses on the enrichment analysis of annotated cancer-related genes in samples with and without changes, so as to find some enriched variants. The Survival page displays the survival curves of the retrieved genes. Overall survival prognosis and disease-free survival prognosis were plotted between tumor samples with at least one variant and tumor samples without variants, and *p*-values and survival times were determined for both groups. This webpage shows a web map analysis integrating data from the Human Reference Protein Database (HPRD), Reactome, National Cancer Institute (NCI)-Nature, and Memorial Sloan-Kettering Cancer Center (MSKCC) cancer cell maps.

### Tissues, Cell Culture and Transfection

This study was approved by the Ethics Committee of the Zhejiang Province’s People Hospital. All specimens were pathologically confirmed as colorectal cancer. No patients received preoperative neoadjuvant radiotherapy or had multiple tumors. Fresh surgical specimens were rapidly frozen in liquid nitrogen and then stored at -80°C. Clinical information of CRC patients is shown in Table 1.

**TABLE 1:** Correlation between clinicopathological characteristics of patients and HMGB3 protein expression.

Variable	HMGB3			
	N	Low expression (*n* = 66)	High expression (*n* = 74)	*p* value
Age (years)				0.193
<65	64	34	30	
≥65	76	32	44	
Gender				0.31
Male	55	23	32	
Female	85	43	42	
TNM Stage				0.001*
I+II	93	54	39	
III+IV	47	12	35	
Depth of invasion				0.009*
T1+T2	14	2	12	
T3+T4	126	64	62	
N stage				0.020*
N0+N1	119	61	58	
N2	21	5	16	
M stage				0.313
M0	133	64	69	
MX	7	2	5	
Differentiation				0.448
Well	114	52	62	
Moderate+poor	26	14	12	
Tumor location				0.327
Right	6	4	2	
Left and rectal	134	62	72	
size				0.104
<5 cm	61	24	37	
≥5 cm	79	42	37	

*p < 0.05.

Human CRC cell lines SW480, RKO, and HCT8 were maintained in our lab and cultured as previously described (Guo et al., 2019). HMGB3 siRNA, scrambled siRNA and OE plasmid were designed and purchased from RiboBio (Guangzhou, China). Lipofectamine^®^2000 Reagent (Invitrogen, Carlsbad, CA, United States) was used for vector transfection according to the manufacturer’s protocols.

### Cell Counting Kit-8 Analysis and Wound Healing Assays

Cell proliferation was detected using Cell Counting Kit‐8 (CCK‐8) assay. CCK‐8 solution (10μL, Dojindo Molecular Technologies, Inc., Kumamoto, Japan) was used for this assay as previously described (Wang et al., 2019). CRC cells were cultured in six-well plates followed by scratching the cell monolayer with a 200 ul pipette tip. Representative images of cell migration were obtained by taking five high-resolution photographs at 0 and 24 h post-damage.

### Western Blot Analysis and Immunohistochemistry

The transfected CRC cells were lysed by protein lysis buffer (Beyotime, Shanghai, China) and the concentrations of protein was measured using a Pierce BCA protein assay kit (Tiangen Biotech Co., Ltd., Beijing, China). A total of 20–30 μg protein was separated by 10% SDS‐PAGE and electro‐transferred to polyvinylidene fluoride membranes (Millipore, Billerica, MA, United States). After being blocked with 5% skimmed milk, the membranes were incubated with HMGB3 (Wuhan Sanying 27465-1-AP) and GAPDH antibodies (ab181602; Abcam) overnight at 4°C. After incubation with the horseradish peroxidase‐conjugated IgG secondary antibodies (Santa Cruz Biotechnology, Santa Cruz, CA, United States). The protein expression levels were detected by ECL reagent (Millipore) and imaged using Amersham Imager 600 from GE Healthcare Life Sciences (Beijing, China). The blots were semi‐quantified by ImageJ software (1.46; National Institutes of Health, Bethesda, MD, United States) Tissue samples were fixed in 4% paraformaldehyde embedded in paraffin, and sectioned. The tissue sections were incubated with HMGB3 primary anti-bodies at 4°C overnight and then incubated with an HRP-conjugated secondary antibody.

### Statistical Analysis

Data were expressed as mean ± SD and analyzed by GraphPad Prism 8.0 (GraphPad Inc., San Diego, CA, United States). Chi-squared test was performed to determine the clinicopathological correlation of HMGB3 expression. Kaplan-Meier curves were used to assess survival outcomes, and correlations were evaluated with Spearman’s correlation coefficients. A two-sided *p* < 0.05 was the threshold of significance.

## Results

### The Expression of HMGB3 in Different Tumors

**FIGURE 1:**
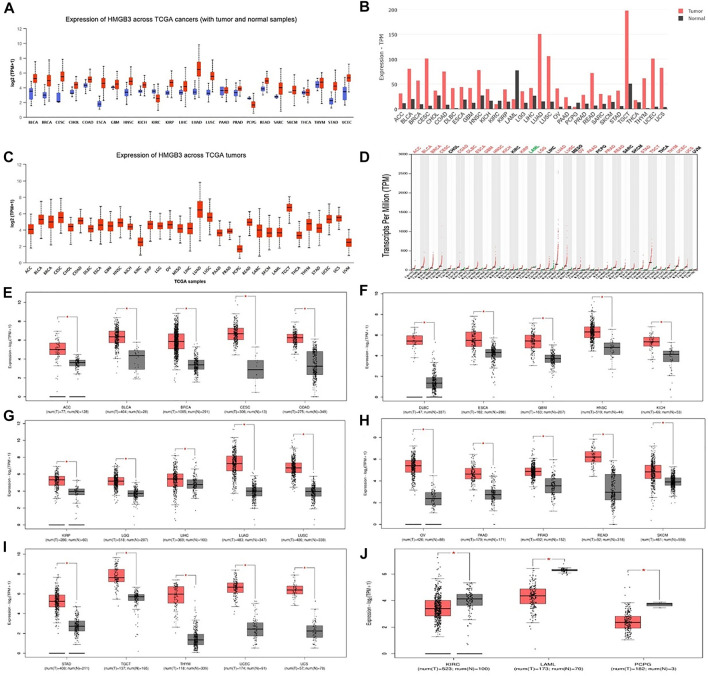
mRNA expression patterns of HMGB3 in different types of human cancer. **(A–C)** Results were analyzed with the Ualcan website. **(B–D)** Results analyzed with the GEPIA2 database. **(E–J)** The expression levels of HMGB3 mRNA in tumor tissues and normal tissues were analyzed based on the GEPIA2 database. *p < 0.05.

**FIGURE 2:**
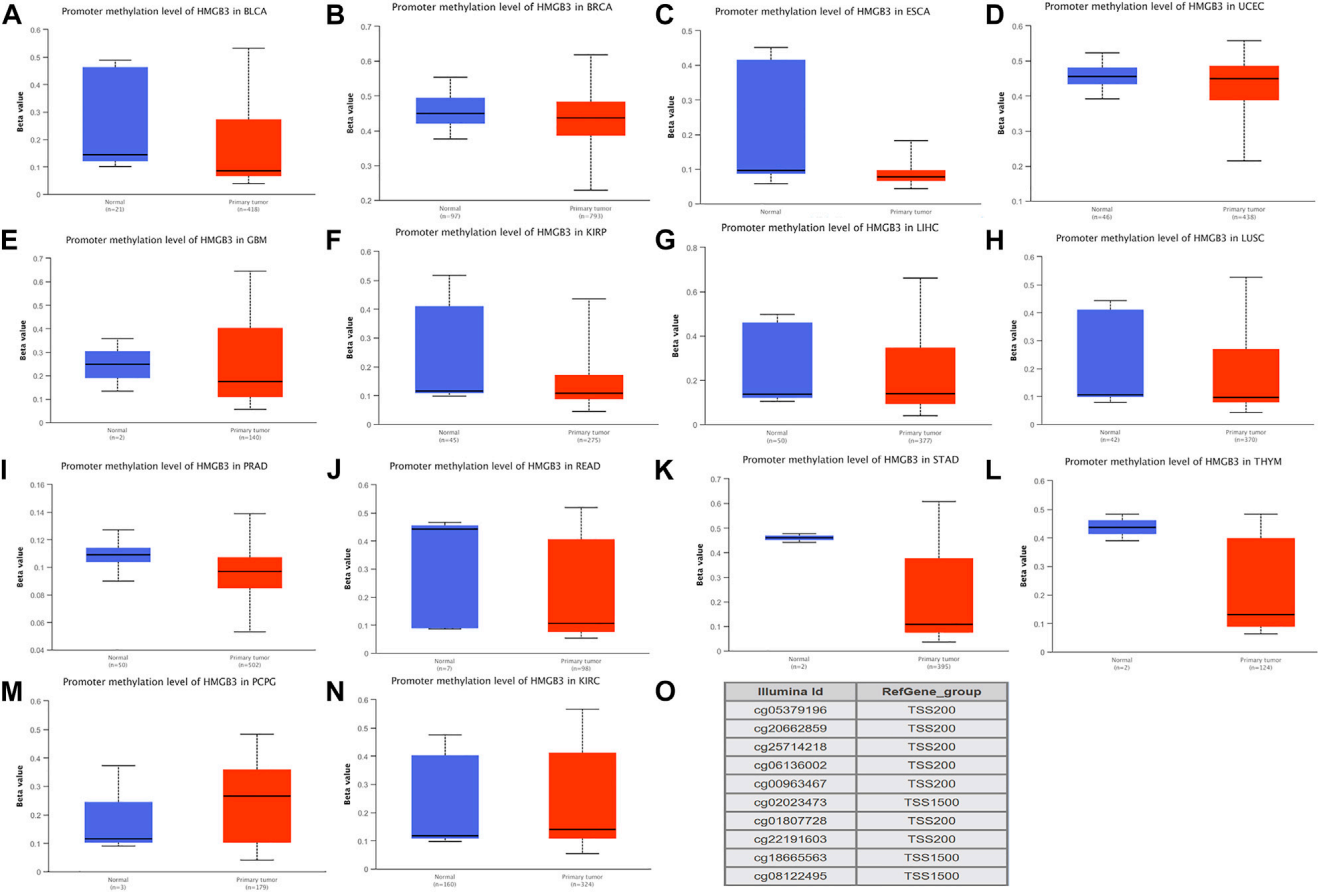
DNA methylation aberration of HMGB3 in tumors.**(A-L)** Twelve highly HMGB3 express tumors presented with decreased DNA methylation level of HMGB3, including BLCA, BRCA, ESCA, UCEC, GBM, KIRP, LIHC, LUSC, PRAD, READ, STAD, and THYM. *p < 0.05. This data was obtained using Ualcan. **(M-N)** Two HMGB3 downregulated tumor, PCPG and KIRC, presented increased DNA methylation level. *p < 0.05. This data was obtained using Ualcan. **(O)** Probes for detecting DNA methylation of HMGB3 promoter.

Analysis of HMGB3 mRNA expression in tumor and normal tissue using the public database Ualcan and GEPIA2. By the Ualcan website, we analyzed the expression of HMGB3 in cancer and normal tissues and found that HMGB3 was highly expressed in most of the tumors (20/24) (Figure 1A, Supplementary Table S1). Similarly, we verified HMGB3 mRNA expression in tumors using an online database (GEPIA2), and the results were consistent with the Uaclan results (Figures 1B,D). In addition, we found that in terms of expression abundance, the highest abundance of LUAD and TGCT were found in all tumors (Figure 1C). In GEPIA2, we further analyzed the expression of HMGB3 in each tumor in detail, and we found that there were 25 tumors in which HMGB3 was highly expressed (Figures 2E–I) and three tumors in which HMGB3 was lowly expressed (Figure 2J). Similarly, we found that HMGB3 was highly expressed in COAD. We explored whether there is a correlation between genetic disorders and HMGB3. We found no correlation between mutations and RNA expression (Supplementary Figure S1A). Also, we found no correlation between DNA copy variants and HMGB3 expression (Supplementary Figure S1B).

### HMGB3 Methylation Analysis

We therefore hypothesized that not all genetic variants cause differential changes in gene expression and that HMGB3 expression is not necessarily due to genetic variation. We then verified the epigenetic disorder of HMGB3.We performed methylation analysis of all HMGB3 high expressing tumors and found that 12 of the 25 HMGB3 high expressing tumors showed hypomethylation in BLCA, BRCA, ESCA, UCEC, GBM, KIRP, LIHC, LUSC, PRAD, READ, STAD, and THYM respectively (Figures 2A–L), while 2 of the 3 HMGB3 low expressing tumors showed hypermethylation in PCPG and KIRC (Figures 2M–N). The DNA methylation level of HMGB3 was subsequently detected using 10 probes located in the HMGB3 promoter (Figure 2O). Subsequently, 13 of the tumors with high HMGB3 expression did not show hypermethylation, and 5 of them had methylation data, but the methylation levels were not changed (Supplementary Figure S2A–E). For the remaining 8 tumors, due to the lack of overall methylation data, we performed methylation level analysis for different tumor stages, and again no changes in DNA methylation levels were found (Supplementary Figure S2F–L).

### Genetic Alterations of HMGB3 in Different Tumor Tissues in cBioPortal

**FIGURE 3:**
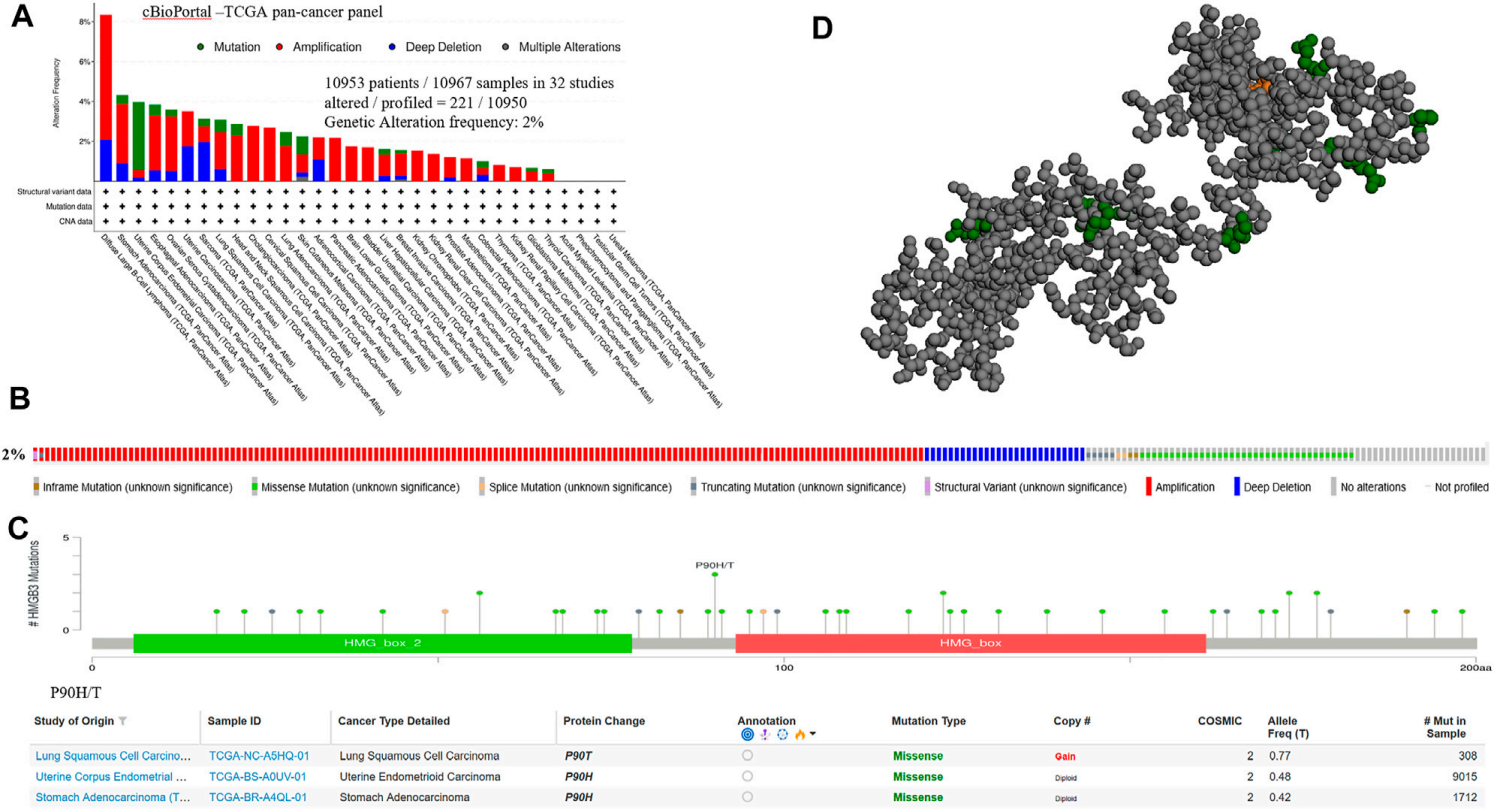
Genetic alterations of HMGB3 in tumor tissues. **(A)** Mutations of HMGB3 in tumor tissues are shown sequentially from top to bottom. **(B)** Frequency of genetic alterations in HMGB3. **(C)** Details of all mutations in tumor. **(D)** 3D structure of P90H/T.

We subjected HMGB3 to a pan-cancer analysis in tumors. Among them, the most common mutations were DNA alterations. tumor mutations of HMGB3 were mainly distributed in UCEC, SCM, and LUAD (Figure 3A). As a result, genetic alterations in HMGB3 occur in 2% of sequenced cases in data obtained from cBioPortal’s OncoPrint schematic (Figure 3B) and the details of all mutations in LUSC, EC and STAD are summarized in Figure 3C. The most common mutation is P90H/T (Figures 3C,D, Supplementary Table S2).

### Correlation of HMGB3 Expression With Prognosis in Different Tumors

**FIGURE 4:**
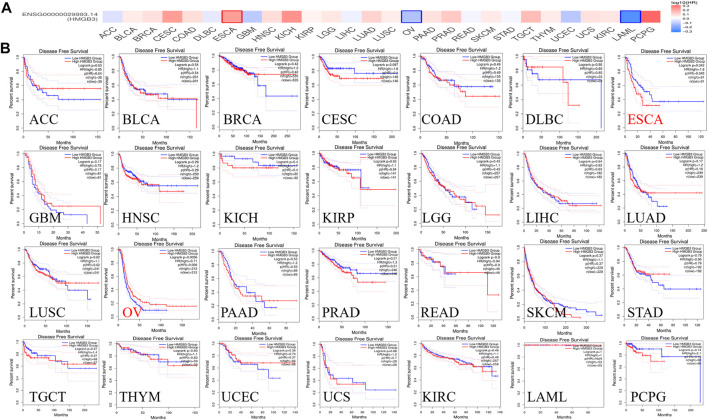
The correlation between HMGB3 gene and DFS data in tumors. **(A)** Survival map in HMGB4 abnormally expressed tumors; **(B)** DFS data in HMGB3 tumors, logrank *p* > 0.05.

**FIGURE 5:**
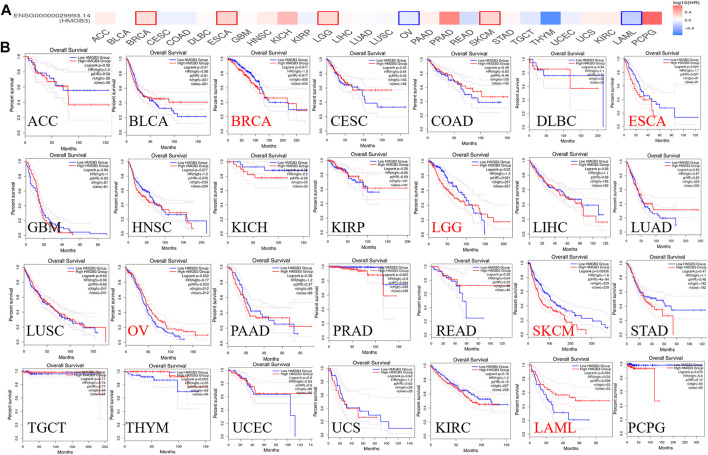
The correlation between HMGB3 gene and OS in tumors. **(A)** Survival map in HMGB4 abnormally expressed tumors; **(B)** OS data in HMGB3 tumors, logrank p > 0.05.

To assess the value of differentially expressed HMGB3 in tumors, we evaluated the correlation between HMGB3 and survival prognosis using GEPIA2. The disease-free survival (DFS) and overall survival (OS) curve is shown in Figure 4, Figure 5 and Supplementary Figure S3, respectively.

Based on DFS analysis, the ESCA patients with low transcriptional levels of HMGB3 was significantly associated with longer DFS (*p* = 0.042) and the OV patients with high transcriptional levels of HMGB3 was significantly associated with longer DFS (*p* = 0.0056) (Figure 4B). Based on OS analysis, the BRCA, LGG, OV, and LAML patients with high transcriptional levels of HMGB3 was significantly associated with longer OS (*p* = 0.017, *p* = 0.02, *p* = 0.032 and *p* = 0.034, respectively), the ESCA and SKCM patients with low transcriptional levels of HMGB3 was significantly associated with longer OS (*p* = 0.0024 and *p* = 0.00036) (Figure 5B). In addition, For DFS, higher HMGB3 expression predicted worse outcome in UVM (*p* = 0.0044) (Supplementary Figure S3C). For OS, higher HMGB3 expression indicated better prognosis in LAML (*p* = 0.0034) (Supplementary Figure S3D). However, higher HMGB3 expression in SARC refers to unfavorable outcome (*p* = 0.012) (Supplementary Figure S3E), which suggests that the therapeutic effect of HMGB3 on patients may need to be customized and individualized, with different treatment regimens for different tumors. However, we evaluated the correlation between HMGB3 gene expression profile and survival in COAD patients. The results showed that there was no significant relationship between OS and DFS of COAD patients and genetic alterations in HMGB3 (Figures 4B, 5B).

### HMGB3-Related Gene Regulatory Networks and Immune Infiltration

**FIGURE 6:**
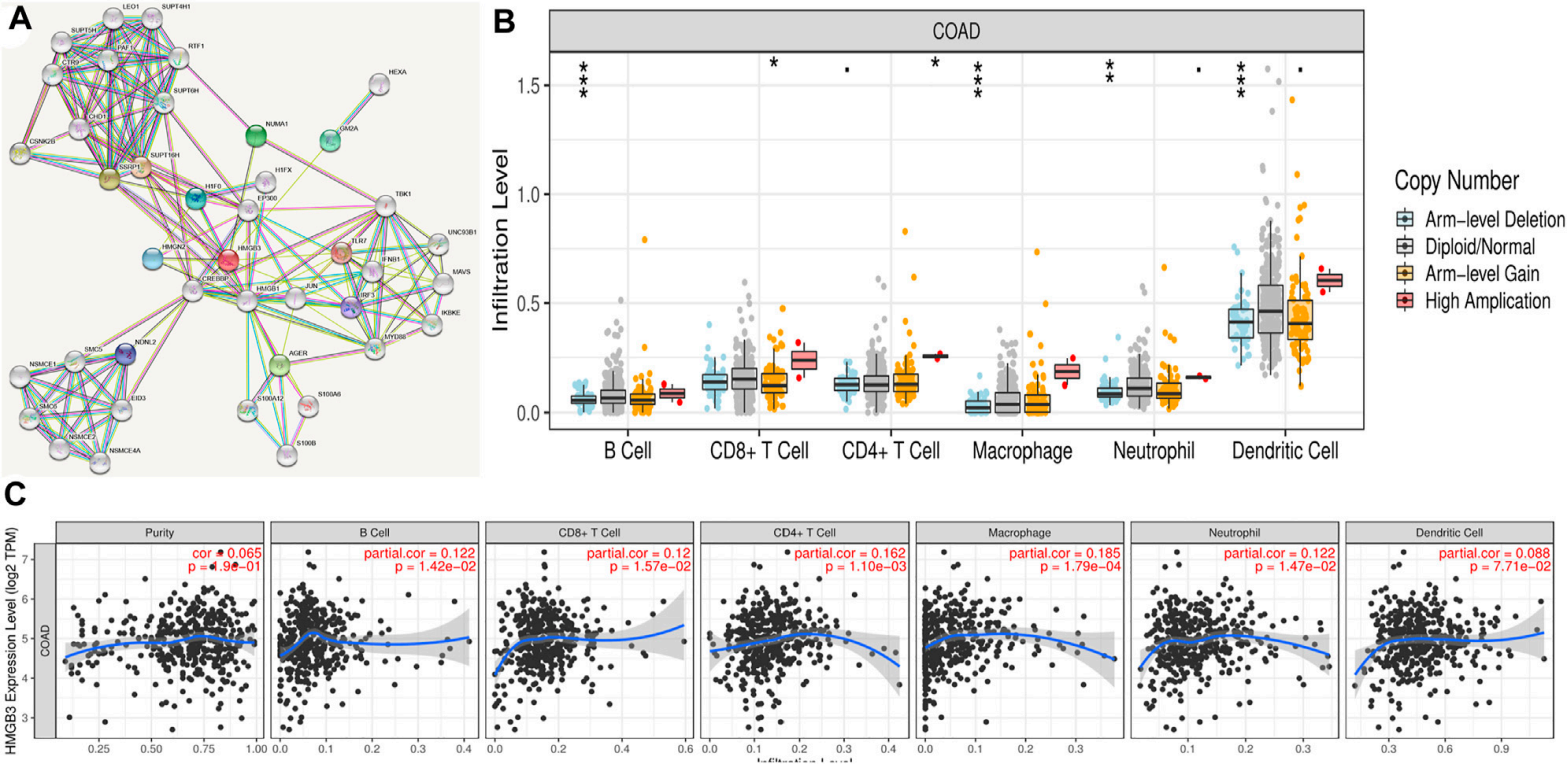
Correlation analysis of the transcript of gene of HMGB3 with the immune infiltration in COAD. **(A)** Gene regulatory network of HMGB3. **(B)** HMGB3 copy number variation affects the infiltration level of CD8+ T cells, macrophages in CRC. *p < 0.05. **(C)** The expression of HMGB3 was no significant relationship with tumor purity, the level of infiltration of CD8+ T cells, CD4+ T cells, macrophages, neutrophils, and dendritic cells in COAD.

To explore the mechanistic role of HMGB3 in tumors, we constructed a gene regulatory network of HMGB3 and neighboring genes. Through string database (https://string-db.org/) we identified SSRP1 and CREBBP as key genes that play a regulatory role in HMGB3 in tumors (Figure 6A). In addition, we performed immuno-infiltration analysis (Figures 6B,C)

### HMGB3 Promotes Proliferation and Migration of CRC Cell Lines *in vitro*

**FIGURE 7:**
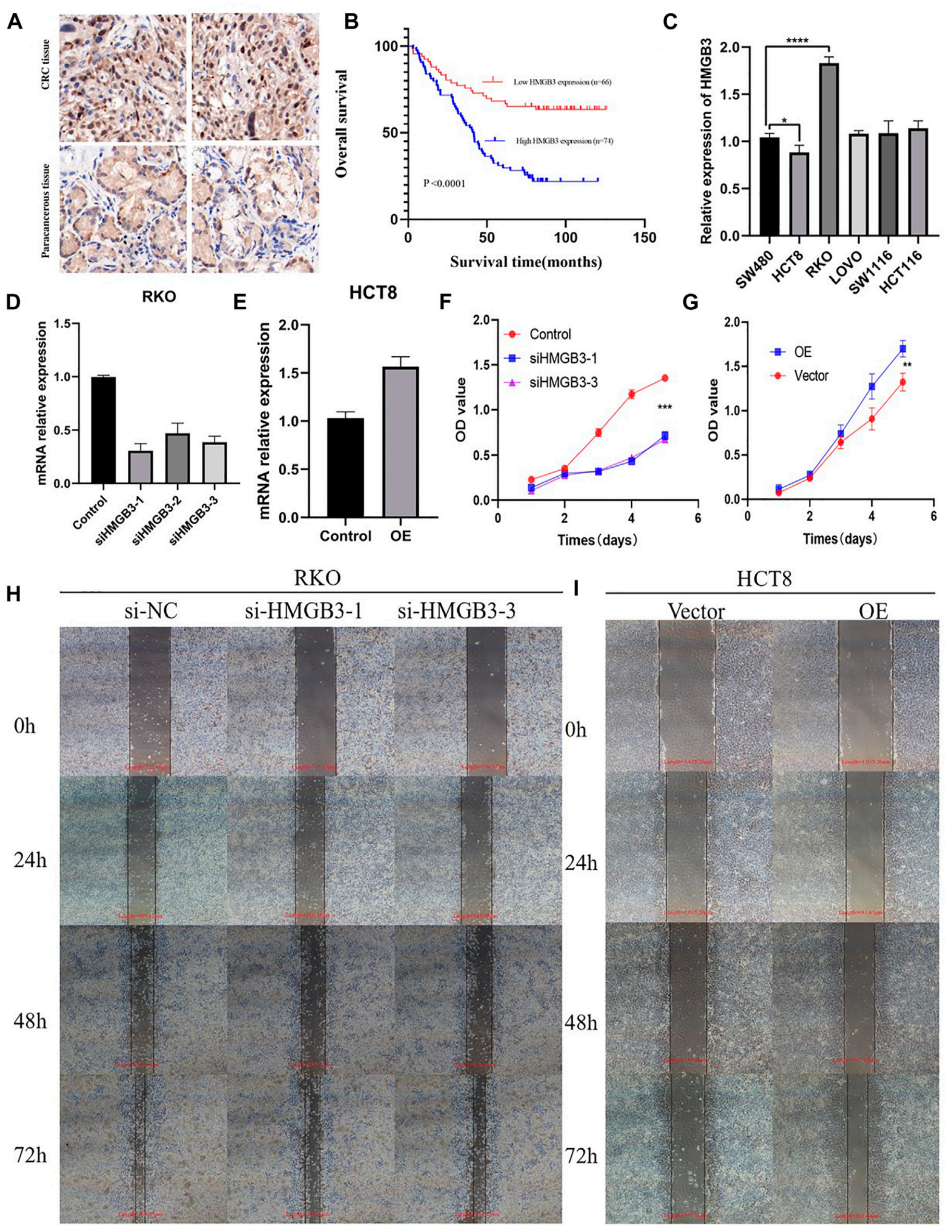
**(A)** CRC tissue and paracancerous tissue samples from TMA were tested by IHC. **(B)** Kaplan-Meier curves with univariate analyses (log-rank) for patients with low HMGB3 expression (red line) versus high HMGB3 expressing tumors (blue line). **(C)** Expression of HMGB3 in colorectal cancer cell lines. There experiments were repeated for three times. **(D,E)** Silencing and overexpression of HMGB3 in colorectal cancer cell lines and detection of HMGB3 expression by qPCR. There experiments were repeated for three times. **(F,G)** Silencing and overexpression of HMGB3 in a colorectal cancer cell line, CCK-8 assay for cell viability. There experiments were repeated for three times. **(H,I)** Silencing of HMGB3 and overexpression of HMGB3 in colorectal cancer cell lines, wound healing assays for cell motility. There experiments were repeated for three times.

We performed further IHC analysis of TMAs containing 140 cases of primary CRC and adjacent normal tissue. The clinicopathological features of these TMAs are listed in Table 1. Positive staining for HMGB3 was stronger in CRC tissues than in adjacent tissues (Figure 7A). Expression of HMGB3 was associated with T stage (*p* = 0.009), N stage (*p* = 0.020), and TNM stage (*p* = 0.001) (Table 1). Kaplan-Meier analysis showed that compared with patients with lower HMGB3 levels, patients with higher HMGB3 levels had poorer OS (*p* = 0.001) (Figure 7B). To investigate the mechanism of HMGB3 in CRC, we designed siHMGB3-1, siHMGB3-2 and siHMGB3-3 of HMGB3, and we also did stable transgenic overexpression, we validated this in six CRC cell lines and showed that HMGB3 was highest in RKO cells and lowest in HCT8 (Figure 7C), so we selected RKO for knockdown and HCT8 cells for overexpression. We then verified by PCR that siHMGB3-1 and siHMGB3-3 worked best in the knockdown group (Figure 7D), so we next used these two knockdown plasmids for deeper validation. The elevated OE plasmid expression abundance in HCT8 cells proved that the construct plasmids were effective Cell (Figure 7E). Counting Kit-8 (CCK8) proliferation assay showed that inhibition of HMGB3 expression levels decreased cell growth (Figure 7F). In contrast, OE showed a higher proliferative capacity compared to Vector (Figure 7G). Wound healing assays were used to determine cell motility, and the results showed that knockdown of HMGB3 impaired the migration of RKO cells (Figure 7H). Correspondingly, overexpression of HMGB3 enhanced the migration of HCT8 cells (Figure 7I).

### HMGB3 Regulates Gene Expression of the WNT/β-Catenin Pathway.

**FIGURE 8:**
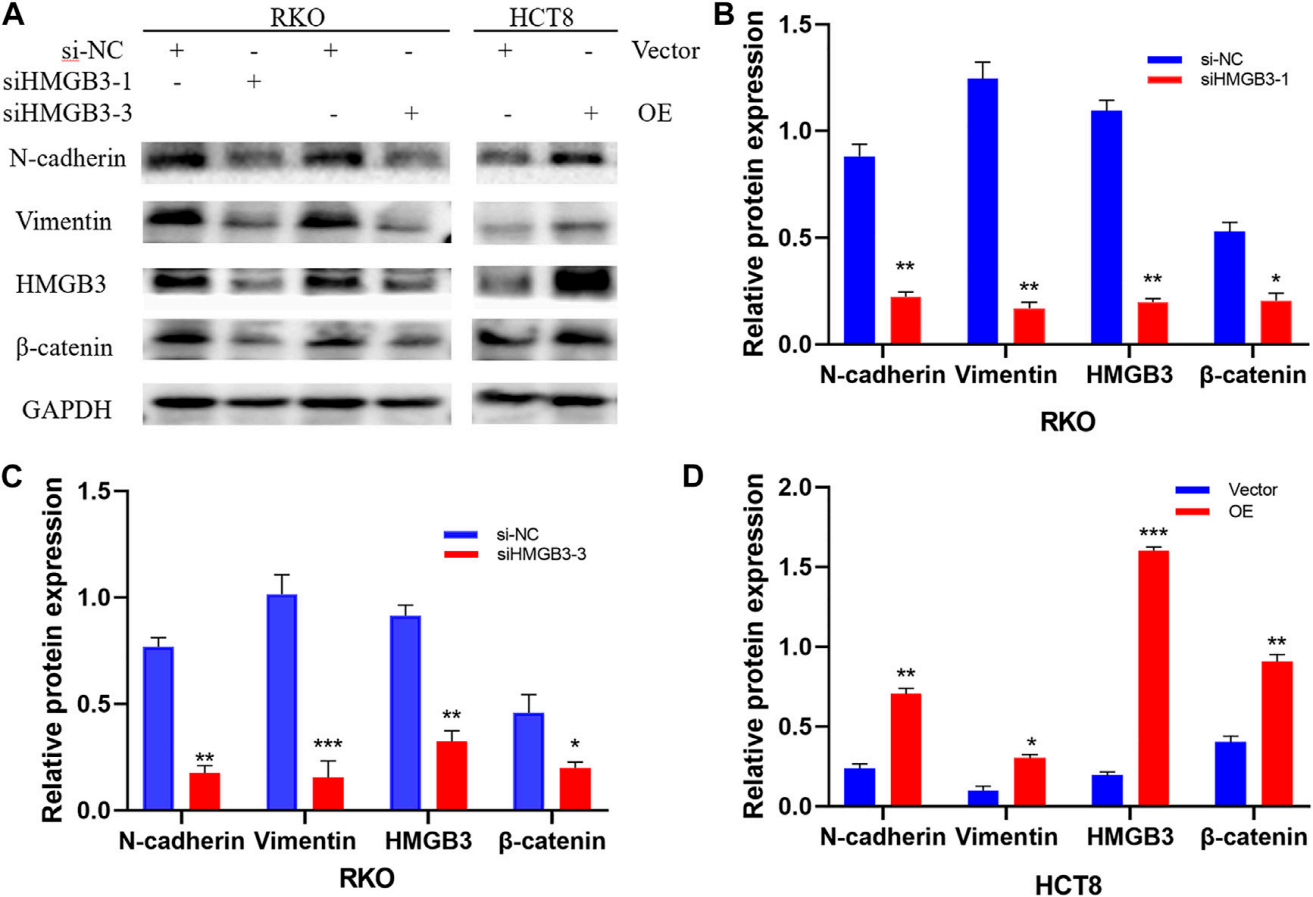
Silencing and overexpression of HMGB3 in colorectal cancer cell lines and WB detection of cellular WNT/β-catenin pathway proteins. There experiments were repeated for three times. **(A)** Western blot was performed to detect N-cadherin, Vimentin, HMGB3, β-catenin and GAPDH expression after Silencing and overexpression of HMGB3. **(B)** N-cadherin, Vimentin, HMGB3 and β-catenin expression after siHMGB3-1 Silencing in RKO cell. **(C)** N-cadherin, Vimentin, HMGB3 and β-catenin expression after siHMGB3-3 Silencing in RKO cell. **(D)** N-cadherin, Vimentin, HMGB3 and β-catenin expression after overexpression in HCT8 cell. GAPDH served as the loading control.

The WNT/β-catenin signaling pathway has an important impact on EMT during tumorigenesis progression. Aberrant activation of this signaling pathway is prevalent in tumor tissues and β-catenin is the central protein of this signaling pathway. To investigate the mechanism of HMGB3 knockdown-mediated colorectal cancer inhibition, we detected that knockdown of HMGB3 decrease mesenchymal markers (N-cadherin, Vimentin) as well as the key factors of WNT/β-catenin signaling pathway (β-catenin). Our research revealed that overexpressing HMGB3 the expression could facilitate EMT and boost WNT/β-catenin signaling pathway which verified by the upregulated expression of β-catenin. Collectively, these results highly demonstrated that HMGB3 possibly promoted EMT process through WNT/β-catenin pathway (Figure 8).

## Discussion

Currently, colorectal cancer is one of the high incidence gastrointestinal tumors. The high mortality rate of colorectal cancer is mainly due to late detection. There are many ways to diagnose colorectal cancer at an early stage, including GI tumor indexes such as CEA, CA125, CA50, and CA724 (Rao et al., 2021), and related imaging examinations such as abdominal CT and pelvic MRI, but each diagnostic method has its own drawbacks. Therefore, it is of great clinical significance to investigate the early diagnostic modalities. Therefore, early screening and diagnosis of colorectal cancer is particularly important. With recent advances in bioinformatics, sequencing and multi-omics technologies have provided more opportunities for early diagnosis of colorectal cancer and prediction of molecular therapeutic targets.

HMG is divided into 3 families, HMGA, HMGB, and HMGN of which the HMGB family has 4 members, namely HMGB1, HMGB2, HMGB3, and HMGB4 (Niu et al., 2020). Research studies have shown that HMGB1 and HMGB2 are closely related to colorectal cancer and can be used as potential diagnostic and prognostic markers (Cheng et al., 2020; Liang et al., 2021). However, HMGB3 and HMGB4 were rarely reported in colorectal cancer (Zhang et al., 2017), so this study analyzed the expression and distribution of HMGB3 in colorectal cancer tissues and analyzed the relationship between HMGB3 gene transcripts and prognosis in conjunction with publicly available databases to explore the biological behavior of the new marker HMGB3 and its potential value for prognostic discrimination.

According to previous studies, HMGB3 can act as an oncogene that promotes tumor growth in gastric cancer (Fang et al., 2020), lung cancer (Xie et al., 2020), laryngeal squamous cell carcinoma (Yuan et al., 2021), bladder cancer (Xie et al., 2019) and glioblastoma (Liu et al., 2018). These studies suggest that HMGB3 can act as a tumor promoter leading to the development of tumors as well as tumor metastasis. A current study reported that HMGB3 was involved in invasive metastasis in CRC (Zhang et al., 2017). However, the specific mechanisms of HMGB3 in CRC tumor development have not been effectively explored. HMGB3 was found to be aberrantly expressed in most cancers and highly expressed in most tumors, including colorectal cancer. To analyze the relationship between HMGB3 and CRC in detail and in depth, we chose the publicly available databases GEPIA2 and Ualcan website. We found that HMGB3 is highly expressed in colorectal cancer in both a state. To explore the relationship of specific clinical index information, we performed a correlation analysis using the survival prognosis of 140 colorectal cancer patients with TMA and found that high expression of HMGB3 had a poor prognosis.

Based on this, we analyzed the role of HMGB3 in tumors by bioinformatics techniques through public databases by pan-cancer analysis, as well as explored the methylation of HMGB3 in different tumors and the immune profile of HMGB3 in colorectal cancer, and verified by qRT-PCR, WB and scratch assays that HMGB3 may play a role in tumor development through the wnt/signaling pathway. invasive role. These results suggest that HMGB3 is associated with CRC disease progression. The current study correlated between tumorigenesis and tumor immune infiltration (Wu et al., 2021). Through public data mining, we also found a correlation between HMGB3 expression and immune infiltration of CRC. Therefore, to investigate the specific tumorigenesis mechanism in depth we performed basic experimental validation.

Our aim was to experimentally verify the expression abundance of HMGB3 in CRC and to explore the function of HMGB3 in tumors. Based on the IHC results suggesting high expression of HMGB3 in tumor tissues, we also showed strong positive bands in WB, thus confirming the high expression of HMGB3 in CRC tissues. We also validated in CRC cell lines and found that the expression was more abundant in RKO cells compared with HC8, so we performed knockdown and overexpression validation by constructing siRNA plasmids as well as overexpression plasmids.

We then performed siRNA plasmid knockdown in RKO cells and HMGB3 gene overexpression in HCT8, and we found that the mRNA abundance was significantly lower in the knockdown group compared to the control group, and on the contrary, the mRNA was significantly higher in the overexpression group. We then performed CCK8 experiments, and again we found that inhibition of HMGB3 expression levels decreased cell growth. Also we did cell scratching assay results suggesting that inhibition of HMGB3 impairs cell migration. To investigate the mechanism of HMGB3 knockdown-mediated colorectal cancer inhibition, we detected a downregulation of N-cadherin, Vimentin and β-catenin proteins after knockdown of HMGB3, and overexpression of HMGB3, we detected a downregulation of N-cadherin, Vimentin and β-catenin proteins were upregulated. These results suggest that knockdown of HMGB3 may reduce CRC proliferation and migration by affecting the expression of these gene proteins.

In conclusion, the present study provides in-depth corroboration for the role of HMGB3 in the development of CRC. We found that HMGB3 can play important roles in colorectal cancer such as proliferation and migration, and can play corresponding roles through the WNT/β-catenin signaling pathway. By inhibiting HMGB3, the migration and proliferation of CRC cells can be effectively reduced. Therefore, HMGB3 can be an effective target for CRC treatment in the future, and we have reason to believe that HMGB3 will be of greater value in more tumors in the near future.

## Data Availability

The original contributions presented in the study are included in the article/**Supplementary Material**, further inquiries can be directed to the corresponding authors.
