# Supplementary material for: HMGB3 is a Potential Therapeutic Target by Affecting the Migration and Proliferation of Colorectal Cancer
Source: Front Cell Dev Biol. 2022 May 31;10:891482. doi: 10.3389/fcell.2022.891482 (PMC9194825; doi:10.3389/fcell.2022.891482)
Supplement: Supplementary file 1 [file DataSheet1.docx]

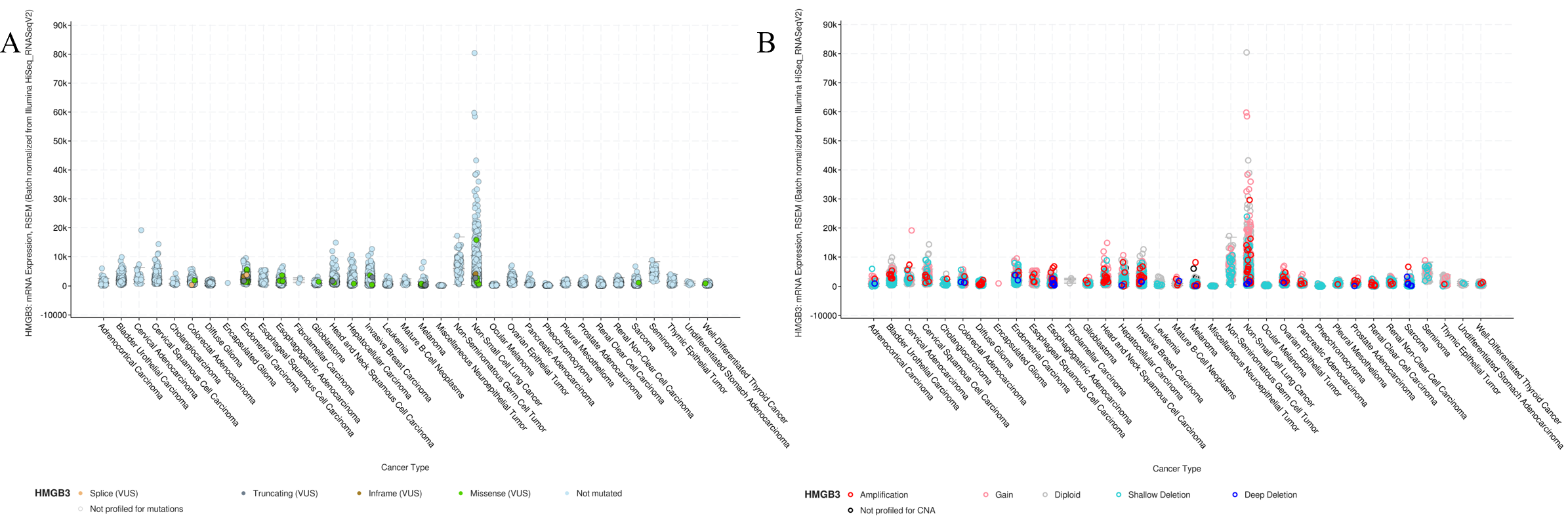


Fig.S1 Correlation between mutations and RNA expression. (A) HMGB3 Mutations (B) Copy number alteration of HMGB3


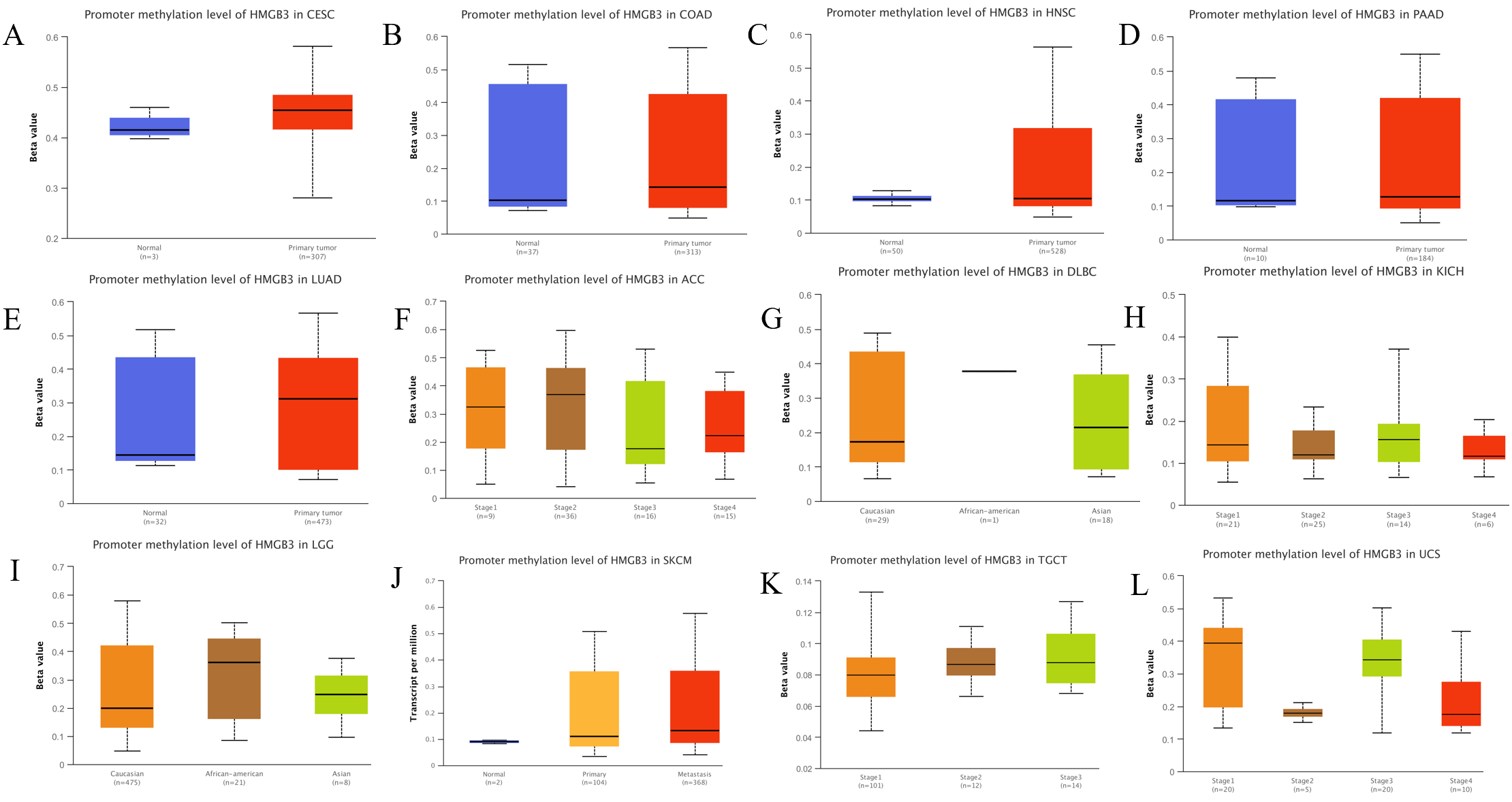


Fig.S2 Correlation between DNA copy variants and HMGB3 expression. (A-E) The methylation levels were not changed. (F-L) Methylation level analysis for different tumor stages and the methylation levels were not changed.


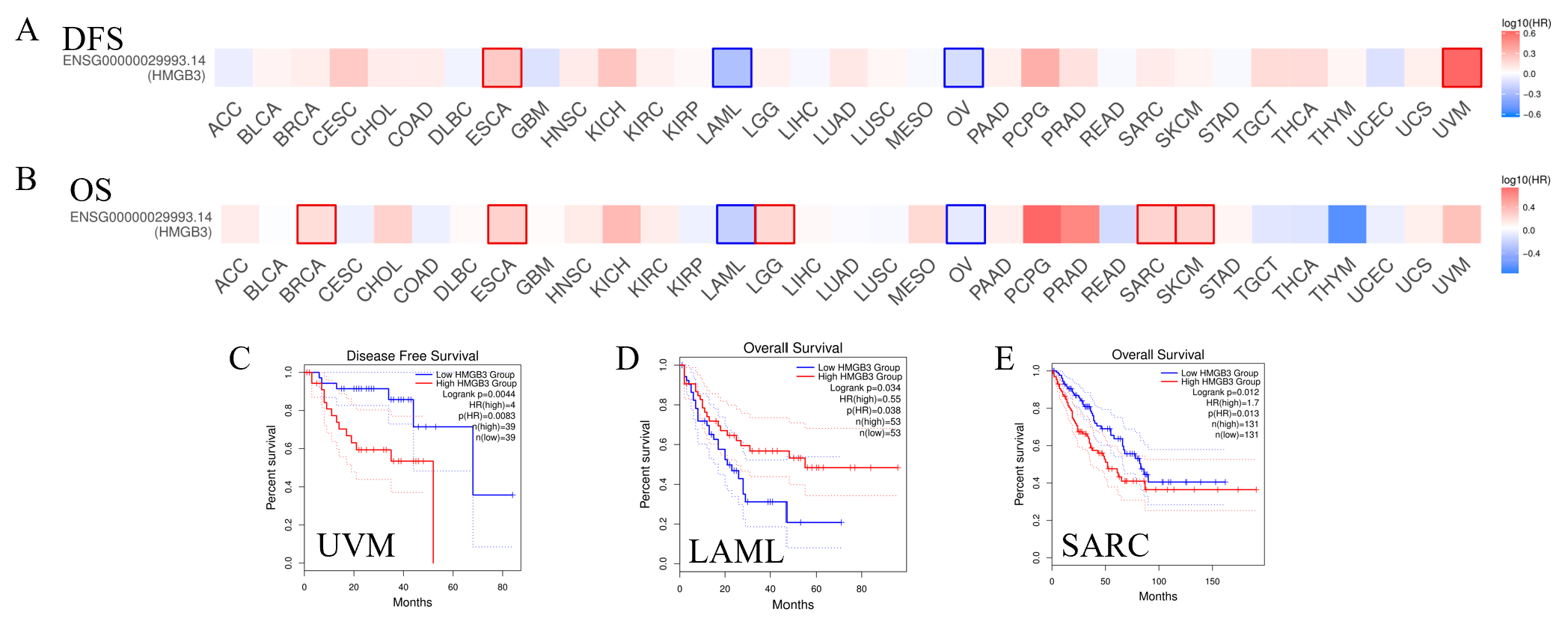


Fig.S3 (A, B) The correlation between HMGB3 gene and survival in tumors. (C) For DFS, higher HMGB3 expression predicted worse outcome in UVM. (D) For overall survival, higher ACE2 expression indicated better prognosis in LAML. (E) For overall survival, higher ACE2 expression indicated worse prognosis in SARC.

Table S1 Mutational profile of HMGB3 in tumor samples.

Table S2 The TPM expression of HMGB3 in different tumors from TCGA database.
